# Does Pilocarpine-Induced Epilepsy in Adult Rats Require *Status epilepticus*?

**DOI:** 10.1371/journal.pone.0005759

**Published:** 2009-06-02

**Authors:** Graciela Navarro Mora, Placido Bramanti, Francesco Osculati, Asmaa Chakir, Elena Nicolato, Pasquina Marzola, Andrea Sbarbati, Paolo Francesco Fabene

**Affiliations:** 1 Department of Morphological and Biomedical Sciences, Section of Anatomy and Histology, University of Verona, Verona, Italy; 2 IRCCS Centro Neurolesi “Bonino-Pulejo”, Messina, Italy; 3 Experimental MRI Center, Faculty of Medicine, University of Verona, Verona, Italy; Universidade Federal do Rio de Janeiro (UFRJ), Instituto de Biofísica da UFRJ, Brazil

## Abstract

Pilocarpine-induced seizures in rats provide a widely animal model of temporal lobe epilepsy. Some evidences reported in the literature suggest that at least 1 h of *status epilepticus* (SE) is required to produce subsequent chronic phase, due to the SE-related acute neuronal damage. However, recent data seems to indicate that neuro-inflammation plays a crucial role in epileptogenesis, modulating secondarily a neuronal insult. For this reason, we decided to test the following hypotheses: a) whether pilocarpine-injected rats that did not develop SE can exhibit long-term chronic spontaneous recurrent seizures (SRS) and b) whether acute neurodegeneration is mandatory to obtain chronic epilepsy. Therefore, we compared animals injected with the same dose of pilocarpine that developed or did not SE, and saline treated rats. We used telemetric acquisition of EEG as long-term monitoring system to evaluate the occurrence of seizures in non-SE pilocarpineinjected animals. Furthermore, histology and MRI analysis were applied in order to detect neuronal injury and neuropathological signs. Our observations indicate that non-SE rats exhibit SRS almost 8 (+/22) months after pilocarpine-injection, independently to the absence of initial acute neuronal injury. This is the first time reported that pilocarpine injected rats without developing SE, can experience SRS after a long latency period resembling human pathology. Thus, we strongly emphasize the important meaning of including these animals to model human epileptogenesis in pilocarpine induced epilepsy.

## Introduction

Temporal lobe epilepsy (TLE) is the most common epileptic syndrome in adult humans (see, for review, [1]); for this reason, the neurobiological bases of TLE have been extensively studied in preclinical research (see, i.a., [1], [2]), and adequate animal models paralleling human pathology are required. TLE refers to a chronic condition characterized by seizures primarily involving the temporal lobe, despite of the fact that other structures, such as the neocortex, may be the origin of the seizures [3]. In rodents, systemic administration of single dose of pilocarpine, a muscarinic cholinergic agonist, lead to *status epilepticus* (SE) and, after a seizure-free period, to a chronic condition determined by spontaneous recurrent seizures (SRS). Pilocarpine has been recently used also to model pharmacoresistance in TLE [4].

Initially, this model has been proposed as sufficiently isomorphic with the human disease [5], but several aspects seem to differ significantly, at least concerning the extent of damage and the incidence of SE, as well as the inflammatory origin [6]. In fact, 2/3 of human patients suffering TLE presents hippocampal sclerosis, whereas the remaining 1/3 presents focal limbic lesions. This latter group does not exhibit pronounced segmental neuronal cell loss or concomitant sclerosis [7]. On the other hand, massive SE-induced neurodegeneration in different brain areas has been reported in pilo-treated animals [8], as well as in other post-SE models of TLE (e.g., kainic acid [9]–[11]). The pattern of the damaged structures is similar in both models, but with different temporal profile of brain injury [9].

It is interesting to note that systemic administration of convulsant dose of pilocarpine (>320 mg/Kg) does not elicit SE in all injected animals [9], [12]. These animals are often repeatedly injected until SE-onset [13], [14] or used as control group [15], [16], whereas no studies are available on possible long-term EEG effects induced by pilocarpine on these animals. Despite of the possible relevance to the human pathology, very few experimental works have focused their attention to this phenomenon, and results seem contrasting. De Mello and colleagues (2005) reported that pilocarpine-injected animals without developing SE are preserved from age-related memory and cognitive impairment [17].

**Figure 1:**
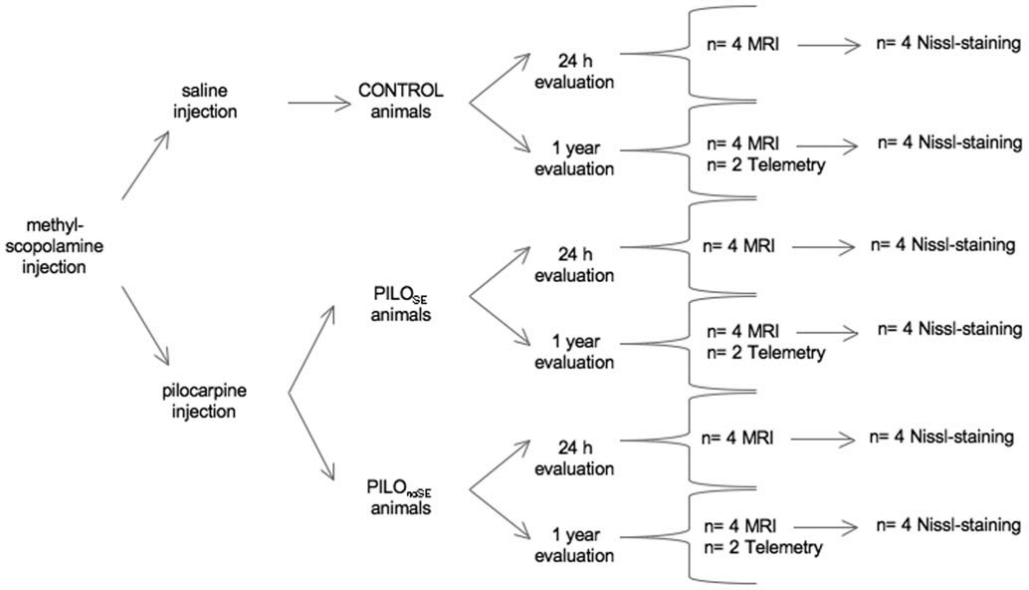
Schematic representation of the experimental design relative to all the different conditions and techniques performed.

This study is aimed to assess: a) whether pilocarpine-injected rats that did not develop SE may exhibit long-term chronic SRS; b) pathological alterations in different time-points in the two experimental groups (SE versus no-SE pilo-injected rats) using structural and functional MRI; c) neuropathological evidences studied by the mean of an accurate stereological study. Therefore, we planned a comparison between animals injected with the same dose of pilocarpine that developed or did not SE, and saline treated rats (Fig. 1).

## Results

### Seizure detection

The occurrence of seizures was evaluated during the first five working days of each month per 12 months. As expected, control rats did not show any seizure during all the evaluated registration time, and on the other hand, since the first evaluation, pilocarpine-treated animals that develop SE (PILO_SE_ animals) developed SRS that were always present until the end of the registration. On the contrary, surprising findings were evidenced in pilocarpine-treated animals that did not develop SE (PILO_noSE_ animals), in which SRS occurred after eight months (+/−2 months) of monitoring EEG and persisted during the whole registration less frequently (0.5–1 seizures/week) than the cyclical pattern observed in PILO_SE_ animals (4–6 seizures/week).

### MRI data

**Figure 2:**
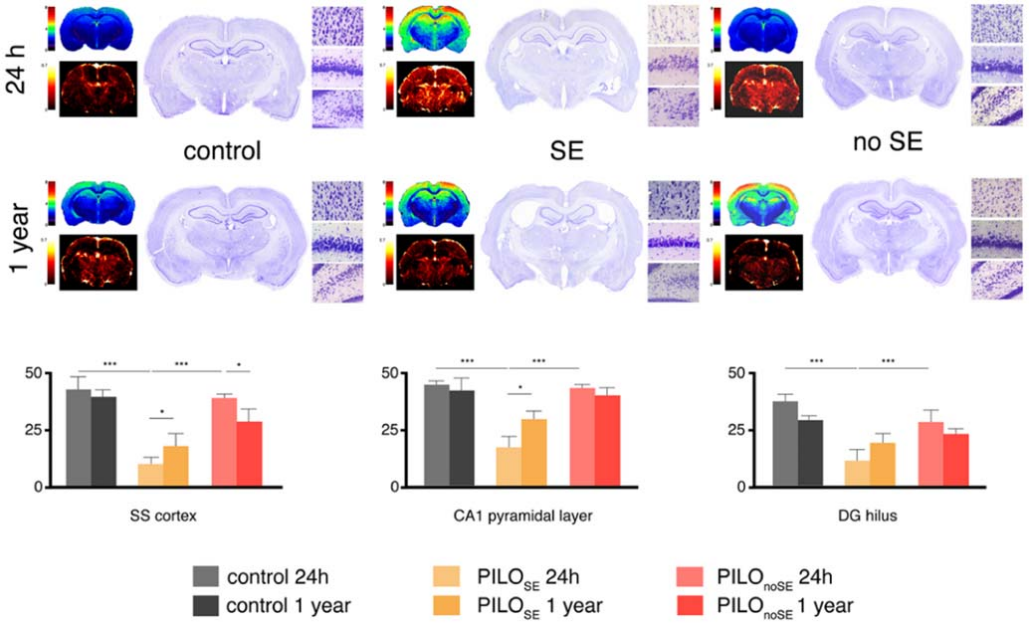
MRI (T2 and rCBV) and histological (low-power vs higher magnifications) data are presented. The three histological areas used for the cell density evaluation reported above are, respectively, cerebral cortex, pyramidal layer of CA1 and hilus of the dentate gyrus.

**Figure 3:**
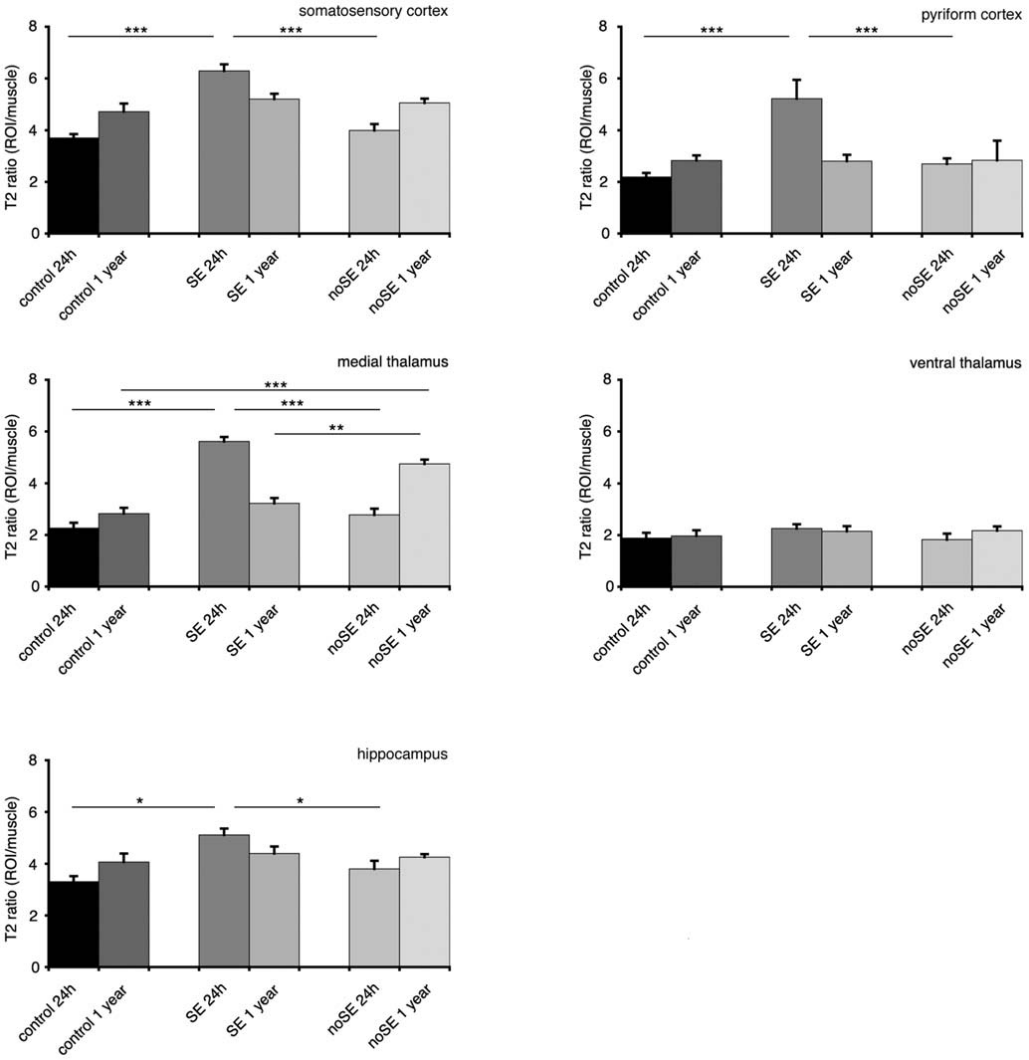
Bar graphs for T2 ratio in different brain areas. T2 ratio was calculated dividing T2 values for each region of interest (ROI) for the baseline (muscle levels). Data were evaluated with one-way analysis of variance (ANOVA), following LSD post-hoc test, setting the significance at p<.05.

T2-weighted (T2-w) signal changes were also consistently observed in the epileptic animals (24 h PILO_SE_, 1 year PILO_SE_ and 1 year PILO_noSE_ groups; Fig. 2). Hyperintense areas were identified in the neocortex, rhinal and piriform cortices, as well as in the hippocampus and anteromedial thalamus. T2-w images analysis clearly showed a similar pattern of changes in the epileptic rats, highlighting the signal increase throughout the cortex, hippocampus and thalamus over other brain structures (Figs. 2–3). Particularly, we found a significant increase of the signal in somatosensory and piriform cortices of PILO_SE_ animals compare to both, control and PILO_noSE_ (p<0.001) 24 h after pilocarpine injection, and the same differences but less noted (p<0.05) were observed in hippocampus at the same time point (24h). But the most interesting result was observed in medial thalamic nuclei, where, although at 24 h the signal was significantly increased (p<0.001) in PILO_SE_ animals with regard to the control and PILO_noSE_ ones, it appeared an opposite signal increasing when evaluating 1 year later the same groups, being highly significant the increase of the signal in the PILO_noSE_ group compare to the control (p<0.01) and PILO_SE_ group (p<0.001).

**Figure 4:**
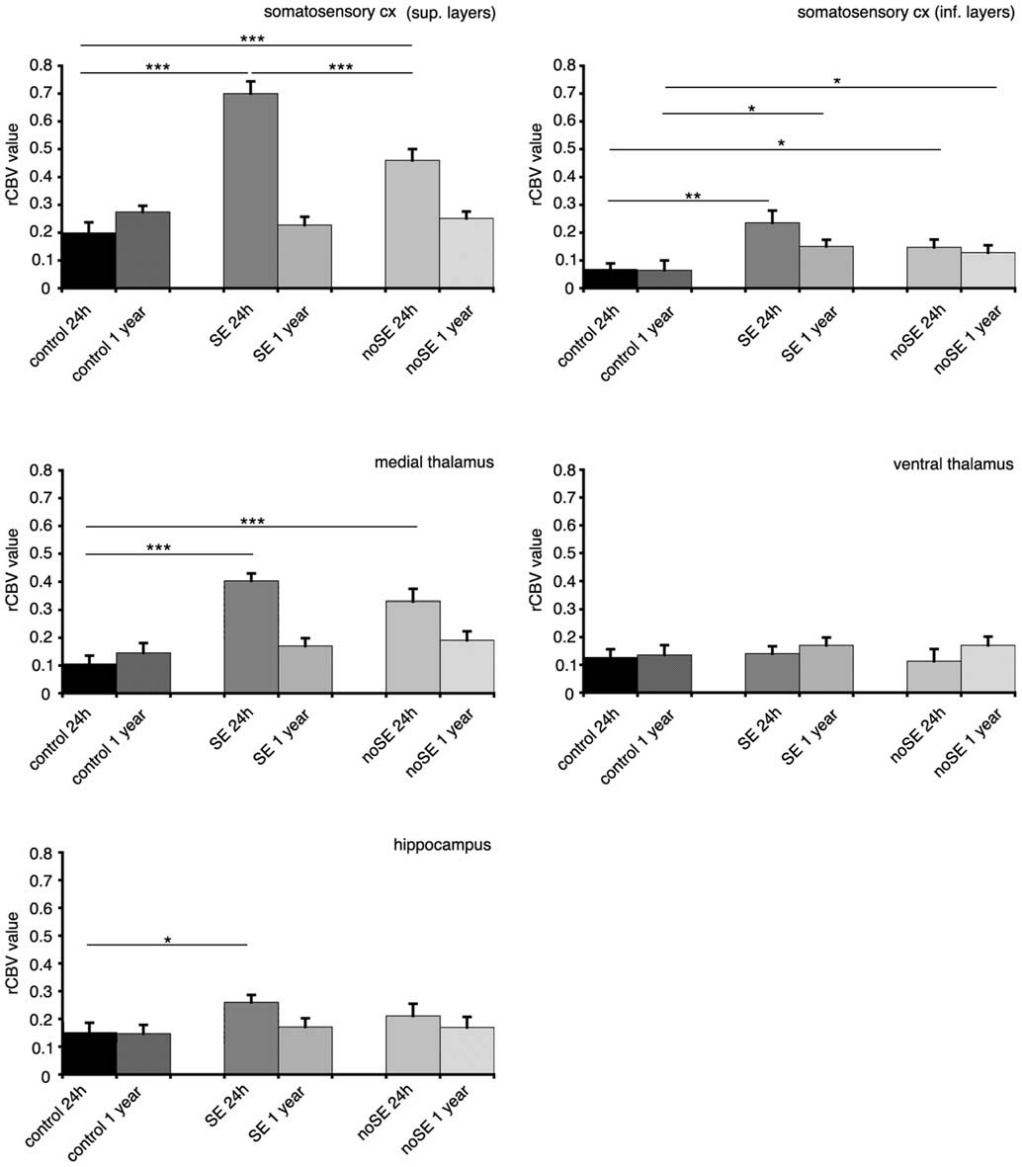
Bar graphs for rCBV values in the same brain areas that were identified for T2 analysis.

Regarding rCBV evaluation, we observed interesting findings in cerebral cortex and thalamus (Fig. 2; Fig. 4). Less marked differences were found in hippocampus. Specifically, only the 24 hours study provides statistically significant results except for the inferior layers of the somatosensory cortex where rCBV value was significantly increased (p<0.05) in PILO_SE_ and PILO_noSE_ animals compare to control ones after 1 year of follow up (Fig. 4). Thus, after 24 h of the pilocarpine/saline injection, the increase of the value was highly significant in superficial layers of the somatosensory cortex for both, PILO_SE_ and PILO_noSE_ animals (p<0.001) compare to control ones, being at the same time equally significant the increase of the value for PILO_SE_ animals (p<0.001) respect to PILO_noSE_ animals. Also in medial thalamus we found significance (p<0.001) for PILO_SE_ and PILO_noSE_ animals compare to control animals and, in hippocampus only PILO_SE_ animals showed higher values (p<0.05) respect the others. It is clear that rCBV results thus in a more predictive and sensible method to detect and forecast future structural and functional alterations that will appear only many months later. Furthermore, these data indicate that PILO_noSE_ animals do not show any structural alterations during the acute phase, whereas vascular alterations are more precocious.

### Histopathological data

Histopathological data were analyzed and compared with MRI data. In Nissl-stained sections, tissue and cell changes in pilocarpine injected animals brains exhibited a closed topographical correspondence with the areas of T2W hyperintensity (Figs. 2–3). In the cerebral cortex of PILO_SE_ animals, edematous alterations were found throughout SS cortex 24 h and 1 year of pilocarpine injection (Fig. 2). In the acute phase (24 h after SE-onset) pyramidal neurons appeared shrunken with a fusiform shape showing a typical picnotic conformation (Fig. 2). Rarefaction of neurons and dilated intercellular spaces were also detected in the hippocampus. In PILO_noSE_ animals non significant alterations have been observed both quantitatively and qualitatively, with no evident signs of neuronal suffering or degeneration.

## Discussion

We hereby report for the first time that animals injected with pilocarpine that did not exhibit SE acutely (PILO_noSE_) developed late-onset SRS many months after pilo injection. Differently to PILO_SE_ animals, SRS can be unrelated to previous acute MRI detectable cellular or tissue damage. However, perfusion MRI demonstrated to be highly sensible to vascular alterations ongoing in the first 24 h after pilocarpine injection. In fact, even in absence of a clear structural degeneration, an augmented cerebral blood volume in cortical and limbic areas of PILO_noSE_ animals has been detected. In addition, we reported evidences that cortico-thalamic domains can be deeply involved in the pathogenesis of pilocarpine-induced epilepsy.

Our data cofirm previous evidences, that long-term neuronal consequences of high-dose cholinergic activation are not necessarily derived from prolonged seizure activation (as seen in SE) [18].

The present data disconfirm that an initial neurodegeneration is the responsible for epileptogenesis in the pilocarpine model. However, there is a wealth of data indicating that SE, with a minimal duration of 30–60 min is required for epileptogenesis [19], [20]; consequently, SRS has mainly been considered as functional response of the abnormal neuronal circuitry subsequent to post-SE brain damage [19], [20]. The necessity of SE in order to establish chronic SRS is a crucial point in the evaluation of the relevance of this experimental model to the human pathology. As recently reported, translation of experimental SE data to the clinical situation should be made with caution [21]. Status epilepticus in animal models has different pathological features in relation to human condition, and the extent of neuronal damage is likely to be overestimated in experimental studies [21]. We should reason that, whereas in pilocarpine model all animals (100%) that develop SE will experience chronic SRS, in humans SE does not necessarily carry an increased risk of consequent chronic epilepsy [22]. In fact, the risk of epilepsy following acute symptomatic SE was reported by Hesdorffer et al. [23] to be less than ½ (42% of cases), 10 years later. These data suggest that, differently from the animal models, in human patient SE cannot be considered a sufficient condition for epileptogenesis. However, there is substantial evidence in humans indicating that the presence of additional risk factors can contribute to greater chance of developing epilepsy in case of more diffuse damage [24], [25]

Concerning MRI, it is interesting that similar T2-w signals were observed between early stages (24 h) of epileptogenesis in PILO_SE_ and long-term (1 year) effects of PILO_noSE_ animals. T2-w increase is usually associated to an increase in interstitial water associated with the development of vasogenic edema (see, as review, [26]); such alteration can be detected several weeks after the initial insult [27]. Seizure-induced neurodegeneration in the medial thalamus has been linked to short- and long-term consequences, such as chronic SRS [28]. The fact that PILO_noSE_ show MD alterations after 1 year of pilocarpine injection, proximal to SRS-onset, suggest that the epileptogenic mechanisms in these animals can be similar to PILO_SE_, but extended for a longer period. It is interesting to note that other brain areas of PILO_noSE_ rats do not show any significant T2-w alterations, pointing out a crucial role of the thalamus. To this regard, evidence in literature suggest that the high metabolic activity detected in this region after 24 h of pilocarpine administration may reflect a sustained increase in inhibitory activity for seizure control, taking part in the chronic epileptic phenomena [29]. Furthermore, the observed cell loss after 1 year in PILO_nose_ would be coherent with the destruction of a structure involved in seizure inhibition [30].

Regional perfusion levels provide critical information on the development of tissue damage [26]. Perfusion-weighted technique has been applied to the study of experimental epilepsy (see, i.a., [31], [32]), indicating a crucial role of vascular alterations in the pathophysiology of pilocarpine-induced epilepsy. Parametrical rCBV maps supply quantitative information on physiological and/or pathological blood volume, providing thus a functional parameter that can be also independent to structural lesions.

Regional CBV alterations can suggest a direct effect of pilocarpine on the endothelial cells. It is interesting to note that vascular alterations, and in particular leukocytes recruitment, have been recently reported to play a key role for epileptogenesis [6]. Furthermore, recent works demonstrated a direct effect of pilocarpine on endothelial cells [33], [34]. In particular, it has been demonstrated that seizure-like activity was obtained in vivo only in case of blood brain barrier (BBB) leakage. Furthermore, Marchi and collaborators reported that focal BBB leakage and increased serum levels of IL-1 β occur in vivo after pilocarpine injection prior to the development of SE and despite of methyl-scopolamine pretreatment [33]. These evidences, all together, point out a peripheral action of systemic pilocarpine injection when inducing epilepsy and could explain the vascular alterations found after 24 hours of the pilocarpine injections in PILO_noSE_ rats.

Our histopathological data are in agreement with Scharfman and collaborators [12]. These Authors used PILO_noSE_ animals for slice experiments from approximately 1 to 7 months after pilocarpine administration, finding in ½ of the animals a small degree of cell loss in the entorhinal cortex, as well as, in one animal, mossy fiber sprouting, usually observed in PILO_SE_ animals during latent period [12].

Regarding the absence of neuronal damage in PILO_noSE_ animals after 24 h of the pilo injection, it would be important to take in account that juvenile rats experiencing SE show no cell death and no recurrent seizures. Whereas, in older rats, the experienced seizures and the acute inflammatory reaction suffered would become chronic and excessive, as reflected by the microglial activation in hippocampus and would be accompanied by seizure-induced cell death and development of SRS [35]. In addition, Brandt and collaborators demonstrated that effective neuroprotection (blockade of cell death) after SE did not prevent the development of SRS in adul rats. This report evidences the distinction and the different contribution in epileptogenesis of two processes, inflammation and neuronal death, that could not be consequent, but subsequent processes [36].

We should also consider that animals that not evolve to SE after pilocarpine injection may have a “natural” lower sensitive for seizures (higher threshold), justifying the long-term epileptogenic required time and their lower frequency of SRS (when compared to PILO_SE_); it becomes thus important to identify the individual susceptibility to electrical alterations in new experiments.

Concluding, in this experiment we have evidences for the first time, that pilocarpine injected rats without developing SE, can experience SRS after a long latency period resembling human pathology. For these reasons, we strong emphasize the importance of including these animals in the study of epileptogenesis modeling human epilepsy. It remains unclear how other mechanisms can contribute to the appearance of chronic seizures in the pilocarpine model, and further investigations are required.

## Materials and Methods

We used 2-moths-old male adult Wistar rats weighing 250–300 g. kept under controlled environmental parameters (temperature 23±3.0°C, humidity 50–60% and 12 h inverted light-dark cycles) and veterinarian control. The experiments were conducted following the principles of the NIH Guide for the Use and Care of Laboratory Animals, and the European Community Council (86/609/EEC) directive.

Animals were injected with methyl-scopolamine (Sigma, 1 mg/kg, i.p) 30 min prior pilocarpine injection (Sigma, 380 mg/kg, i.p) to minimize peripheral cholinergic effects. Control rats received saline instead of pilocarpine. Immediately, rats were video recorded for 24 hours and during this time, or until 15 days after, depending on the SE occurrence, pilocarpine injected animals were subdivided in SE (PILO_SE_) or non-SE (PILO_noSE_) groups (see [10]). Four hours after pilocarpine/saline injection, all animals received diazepam administration (single 4 mg/kg i.p. injection) in order to standardize the duration of seizure activity in SE animals. In all groups, the time points investigated were 24 h and 1 year after pilocarpine/saline injection to correlate short and long-term effects.

The experimental design has been summarized in Fig. 1. Two animals belonged to each group were followed up for 12 months by telemetry system as previous studies [5]. After two weeks of the pilocarpine/saline injection, animals were implanted i.p. with the telemetry transmitter TA11CTA-F40 (Data Science International, Arden Hills, MN, U.S.A) for cortical sub-dural above the parietal cortex. EEG recording, locomotor activity and temperature monitoring. These devices continuously sense, process and transmit information from the animal to a data storing system. Prior to the surgery all animals received medetomidine (Dormitor® 0.05 mg/kg s.c) as sedative 5 min before the anesthetic tiletamina+zolacepam (Zoletil® 20 mg/kg i.p), then the analgesic carprofen (Rimadyl® 5 mg/kg s.c), and amoxicilina (Clamoxyl® LA 150 mg/kg s.c) as prophylactic antibiotic. The same dose of the antibiotic was given the day after the surgery. Animals were allowed to recover for one week after surgery. Four equivalent non-implanted rats per group were subjected to MRI technique and Nissl staining to detect morphological features correlated to the data monitoring (Fig. 1).

In the acute study (24 h after pilocarpine-injection), MRI and histological techniques were used to describe neurophatological signs in the groups analyzed. Hyperventilation was observed during SE but ended after its arrest, and all animals exhibited normal spontaneous breathing at the time of MRI analysis. MRI technique has been widely used in studies of experimental epilepsy. MRI experiments were performed using a Bruker Biospec Tomograph equipped with an Oxford, 33-cm-bore, horizontal magnet operating at 4.7T. The data are analyzed with T2W RARE sequences. Animals were anesthetized with 1% halothane in 1 L of oxygen in air per minute (initial dose: 4% halothane); rectal temperature and heart rate were monitored and were similar in control, PILO_SE_ and PILO_noSE_ animals.

Regional cerebral blood volume (rCBV) mapping was carried out using the same Biospec system used structural MRI analysis. In these animals, anesthetized as indicated above, the tail vein was catheterized for contrast medium administration. Sinerem®, an ultra small particle iron oxide (USPIO) contrast agent (kindly supplied by Guerbet, Aulnay-Sous-Bois, France) was used. Sinerem® has an iron-oxide core of about 6 nm diameter coated by dextran (coated particle dimensions of about 20 nm) and is characterized by a blood half-time longer than 2 h in rats [12]. Sinerem® was dissolved in saline and injected at a 6 mg/kg dose (mg are referred to iron) and images were acquired with gradient-echo sequence before and after USPIO administration.

At the end of MRI analysis, the anesthesia was implemented with barbiturates (Tionembutal, 50 mg/kg, ip) and the rats were perfused via the ascending aorta with PBS followed by 4% paraformaldehyde in 0.1 M phosphate buffer, pH 7.4 (PB). Brains were then removed from the skull, left in 30% sucrose overnight and coronal cut (40 μm thickness) on a freezing microtome (Reichert-Jung, Vienna, Austria). All sections were collected, divided in 3 adjacent series and mounted on 2% gelatin-coated slides. The first series were Nissl-stained, dehydrated, and cover slipped. Nissl-stained sections were examined under a microscope under bright-field illumination. The rat atlas of Swanson (1992) was used to match in all animals the same levels of sections selected for further analysis [11].

Histological data (neuronal density) and MRI data (T2W and rCBV values) obtained in the three experimental groups, were analyzed with one-way analysis of variance (ANOVA), followed by the LSD post hoc test, setting the significance at P<0.05.
